# Metal-Polymer Complexes of Gallium/Gallium-68 with Copolymers of N-Vinylpyrrolidonewith N-Vinylformamideand N-Vinyliminodiacetic Acid: A Hint for Radiolabeling of Water-Soluble Synthetic Flexible Chain Macromolecules

**DOI:** 10.3390/polym12122889

**Published:** 2020-12-02

**Authors:** Nikolay I. Gorshkov, Аndrey Yu. Murko, Iirina I. Gavrilova, Мarina А. Bezrukova, Аlbert I. Kipper, Sergei V. Shatik, Аlexander V. Tokarev, Valerii D. Krasikov, Еvgenii F. Panarin

**Affiliations:** 1 Federal State Budgetary Institution of Science Institute of Macromolecular Compounds, Russian Academy of Sciences (IMC RAS), Russian Federation, V.O. Bolshoy pr. 31, 199004 Saint Petersburg, Russia; nitrobenzene@yandex.ru (A.Y.M.); lenchrom@hq.macro.ru (I.I.G.); bezrukova@imc.macro.ru (M.A.B.); kipper@imc.macro.ru (A.I.K.); krasikov@lenchrom.ru (V.D.K.); panarin@hq.macro.ru (E.F.P.); 2 Federal State Budgetary Institution “Russian Research Center for Radiology and Surgical Technologies” of the Ministry of Health of the Russian Federation, Russian Federation, p. Pesochny, ul. Leningradskaya, 70, 197758 Saint Petersburg, Russia; shatik@hotmail.com (S.V.S.); tatjana.i.tokareva@mail.ru (A.V.T.)

**Keywords:** water-solublepolymer complexes, N-vinylpyrrolidone, N-vinylamine, iminodiacetic acid, metal-polymer complexes, gallium-68

## Abstract

Copolymer of N-vinylpyrrolidone (VP) with vinylformamide (VFA) and N-vinyliminodiacetic acid (VIDA) was synthesized; its metal-polymer complexes (MPCs) with gallium were obtained. The complexes were characterized by size exclusion chromatography, hydrodynamic and optical methods, scanning electron microscopy, and spectral methods (UV, IR, ^1^Н NMR spectroscopy). It was demonstrated that in going from polymer to complex, hydrodynamic parameters of macromolecules change only slightly, although the polymer contains intramolecular Ga(VIDA)_2_ fragments in its structure. A new method for preparation of MPCs with gallium and gallium-68 radionuclide was suggested. The obtained metal-polymer complex is stable over a wide range of pH values as well as in the histidine challenge reaction. In vivo distribution experiments in intact animals showed high primary accumulation of thegallium-68 MPC in blood with subsequent excretion via urinary tract.

## 1. Introduction

Nuclear medicine is a modern non-invasive method for diagnostics and therapy of various pathologies, including oncological diseases. The method consists in labeling of biologically active molecules with radioactive isotopes that have an affinity for target living tissues. In medical practice (diagnostics and therapy), along with biogenic radioactive isotopes (^11,14^С, ^15^N, ^18^F), metal isotopes are widely used (technetium-99m, gallium-68, rhenium-188, indium-111, yttrium-90, etc.) [1,2,3]. Biologically active compounds with low molecular masses (for instance, short peptides) are employed as isotope carriers; however, introducing bulky chelating agents and heavy metal ions into their structure may result in disturbance of their affinity parameters.

Protein aggregates with high molecular weights, such as monoclonal antibodies labeled with radionuclides, are common in clinical practice as site-specific tumor targeting agents, since each of these species possesses high affinity for a given tumor [4]. At the same time, obtaining highly specific antibodies, their isolation, purification, and radiolabeling protocols involve complicated and costly procedures.

A large number of publications are devoted to introducing radionuclides into nanoparticles (gold particles, particles of metal oxides, hypercrosslinked polymers) [5,6,7]. These objects demonstrate prolonged time of circulation in blood and are capable of passive accumulation in target tumors due to an enhanced permeation and retention (EPR) effect [8]. However, there is an unsolved problem associated with clearance of these particles from organs after completion of radionuclide transport [9].

Due to these considerations, the latest research efforts have been concentrated on radiolabeling of dendrimers, synthetic liposomes, micelles, hyperbranched polymers, etc. [10,11,12,13,14], which generally do not suffer from the abovementioned drawbacks. In order to achieve the maximal accumulation at tumor sites, these macromolecules should demonstrate prolonged circulation time in blood, and should not be accumulated in non-target organs in noticeable amounts; they should have controlled dimensions (for effective localization in cells), and be easily cleared from the organism. Нerewith, flexible chain synthetic water-soluble polymers can be considered as promising objects for targeted radionuclide transport [15,16]. For example, mannosylateddextrans decorated with diethylenetriaminepentaacetic acid (*DTPA*) chelation units and labeled with ^99m^Tc are adopted in clinical practice for visualization of defeated lymph nodes under the trade name “Lymphoseek”^TM^ [17].

Since the polymers with attachedchelators can be considered as multi-site chelation systems, metal atom coordination is defined not only by principles of coordination chemistry, but also by structural, sterical, hydrodynamical, and molecular weight characteristics of a polymeric carrier [18].

N-vinylamides belong to the class of flexible chain polymers and can also be regarded as promising polymeric carriers for radioactive isotopes. These polymers have been comprehensively studied and are used in clinical practice as plasma substituents and transporters of low molecular weight biologically active compounds [19,20,21].

Radiolabeling is performed using bifunctional chelating agents (BFC); as a rule, these are macrocycles that create a stable environment around a metal ion and prevent its interaction with reactive donor groups present in biologically active media [22].

Coordination chemistry of the radioactive isotope gallium-68 (T_1/2_ = 68 min, E_β_^+^_max_ = 2.92 МeV) is thoroughly studied [23]; this isotope is widely used in imaging of tumors with positron emission tomography (PET).

The goals of the present work included the synthesis of N-vinylpyrrolidone(VP)/N-vinylformamide (VFA) copolymers containing N-vinyliminodiacetic acid (VIDA) as a bifunctional chelating agent (VP-VFA-VIDA), characterization of the copolymers by spectroscopic and chromatographic methods, study of complexation between the copolymer and gallium ion, isolation of metal-polymer complexes (MPC) (VP-VFA-VIDA)-Ga, estimation of their stability at various pH values and in histidine challenge reaction (HCR), determining conditions of radiochemical synthesis, and evaluation of biological distribution of radiolabeled MPC in intact laboratory animals.

## 2. Experimental

### 2.1. Materials

N-vinylpyrrolidone (*N*-VP, “Sigma-Aldrich”, St Louis, MO, USA) and *N*-vinylformamide (*N*-VFA, Sigma-Aldrich, St Louis, MO, USA) monomers, and 2,2′-azobisisobutyronitrile initiator (AIBN, Biolar, high purity grade, St Petersburg, Russian Federation) were used. The used solvents and reactants had reagent and analytical purity grades and were purchased from Vekton (St Petersburg, Russian Federation) and Sigma-Aldrich (St Louis, MO, USA). 

*N*-VP and *N*-VFA monomers were purified by distillation under vacuum (b.p. = 69 °C (3 mm Hg), n_D_^20^ = 1.5120; b.p. = 65 °C (4 mm Hg), n_D_^20^ = 1.4920, respectively). AIBN initiator was purified by recrystallization from ethanol:chloroform mixture (3:1), m.p. = 103 °C.

Phosphate-buffered saline (PBS) solutions were prepared by dissolving the preformulated tablets (Sigma-Aldrich, St.-Louis, MO, USA)) in 200 mL of Milli-Q water (mean resistivity > 18.2 Ω) to give [NaCl] = 0.138 M, [KCl] = 0.0027 M, and pH 7.4.

### 2.2. Instruments and Measurements

H and ^13^C NMR spectra were recorded using a BrukerAvance II-500 WB spectrometer in deuterated solvent (D_2_O) purchased from Sigma-Aldrich, (St.-Louis, MO, USA). Chemical shifts were measured against the signal of residual non-deuterated solvent (water, 4.8 ppm) and the signal of external tetramethylsilane standard.

IR spectra were measured using a Shimadzu Prestige FTIR spectrometer (in KBr pellets).

UV spectra were registered using a Shimadzu UV-1280 spectrophotometer.

Elemental analysis (C, H, N) was performed using a Vario EL-III elemental analyzer. The percentages of carbon, hydrogen, and nitrogen were estimated.

Chromatographic analysis was performed with the use of a Smartline HPLC instrument (Knauer, Geretsried, Germany) equipped with a JetStream column thermostat, refractometric and spectrophotometric detectors (K-2501 diode array detector, λ = 200–500 nm). Registration of chromatograms and calculations of molecular masses and other parameters were performed using ClarityChrom GPC/SEC V.2.6 xx (Geretsried, Germany). An ultrahydrogel linear SEC column (7.8 × 300 mm) with a pre-column (0.6 × 40 mm, Waters, Milford, MS, USA) was used for analysis of the copolymers. Analyses were carried out in aqueous solution of 0.2 M NaCl as an eluent at 25 °C. Calibration dependences for the columns were plotted using the data for the previously characterized poly(*N*-vinylformamide) standards in 0.2 M aqueous solution of NaCl; the values of the Kuhn–Mark–Houwink constants were K = 10.74 × 10^–3^ and α = 0.76 ± 0.04 [24]. Ultrashort monolith CIM^TM^ (Convection Interaction Media) columns (CIM disks, 1.2 × 0.5 cm) (Ajdovščina, Slovenia) were used for analysis of polymer-metal interaction and for evaluation of radiochemical yield in linear gradient (water-0.01 M HCl).

Intrinsic viscosity [η] was measured using an Ubbelohde viscometer, (Vecton, St.-Petersburg, Russia). Relative viscosity [η_r_] was calculated as an initial slope of the ln(η_r_) = f(c) dependence, i.e., in the region where η_r_ is the relative viscosity of a solution at concentration *c*. The measurements were performed in 0.1 M solution of sodium acetate at 25 °C.

*Translational diffusion coefficients D* were determined at 24 °C; the technique involved the recording of dispersion of the solution–solvent boundary using the Tsvetkov polarizing diffusometer. The images of interference fringes of the solution–solvent boundary were processed using the maximum ordinate method and the method involving measuring areas under interference fringes [25].

Sedimentation coefficients *s* of macromolecules were measured at 24 °C using a MOM 3180 (Budapest, Hungary) equipped with a polarization interferometer [26], at a rotation speed of 40 × 10^3^ rpm. During measurements of diffusion and sedimentation coefficients, concentrations of the solutions did not exceed 0.15 g/dL.

The results of sedimentation and diffusion experiments were used to determine molecular masses of copolymers by the Svedberg method, according to the relationship *M*_sD_ = (*s/D*) × *N*_A_*kT*/(1 −$\bar{\upsilon }-\;\rho o$), where *k* is the Boltzmann constant, and *T* is the absolute temperature. The partial specific volume of copolymer ($\bar{v}$) was calculated additively using mass densities ($\bar{v}$^−1^) of the components (poly(*N*-vinylpyrrolidone) and poly(N-vinylformamide) [27]), taking into account copolymer composition. The value for the copolymer was found to be *v* = 0.775 cm^3^/g.

*Hydrodynamic radii* (*R*_h_) were determined by dynamic light scattering (DLS) with the help of a Photocor Complex correlation spectrometer (light source: a coherent He/Ne laser, power output 20 mW, wavelength λ= 632.8 nm) equipped with a PhotocorFC programmable correlator (288 channels, ZAO Anteks, Russia). The correlation function was processed using Dynals(V.2.1) (Gelios, Russia). This software program allows for the calculating of equivalent sphere hydrodynamic radius *R*_h_ on the basis of the measured diffusion coefficients *D*, according to the Einstein–Stokes equation: *R*_h_ = *kT*/6π*Dη*_s_, where *η*_s_ is the solvent viscosity, and *T* is the temperature.

### 2.3. Synthesis of VP-VFA and VP-VAcopolymers

The starting monomers (VP and VFA, Aldrich) were purified by vacuum distillation at 10−2 ppm.

**Scheme 1:**
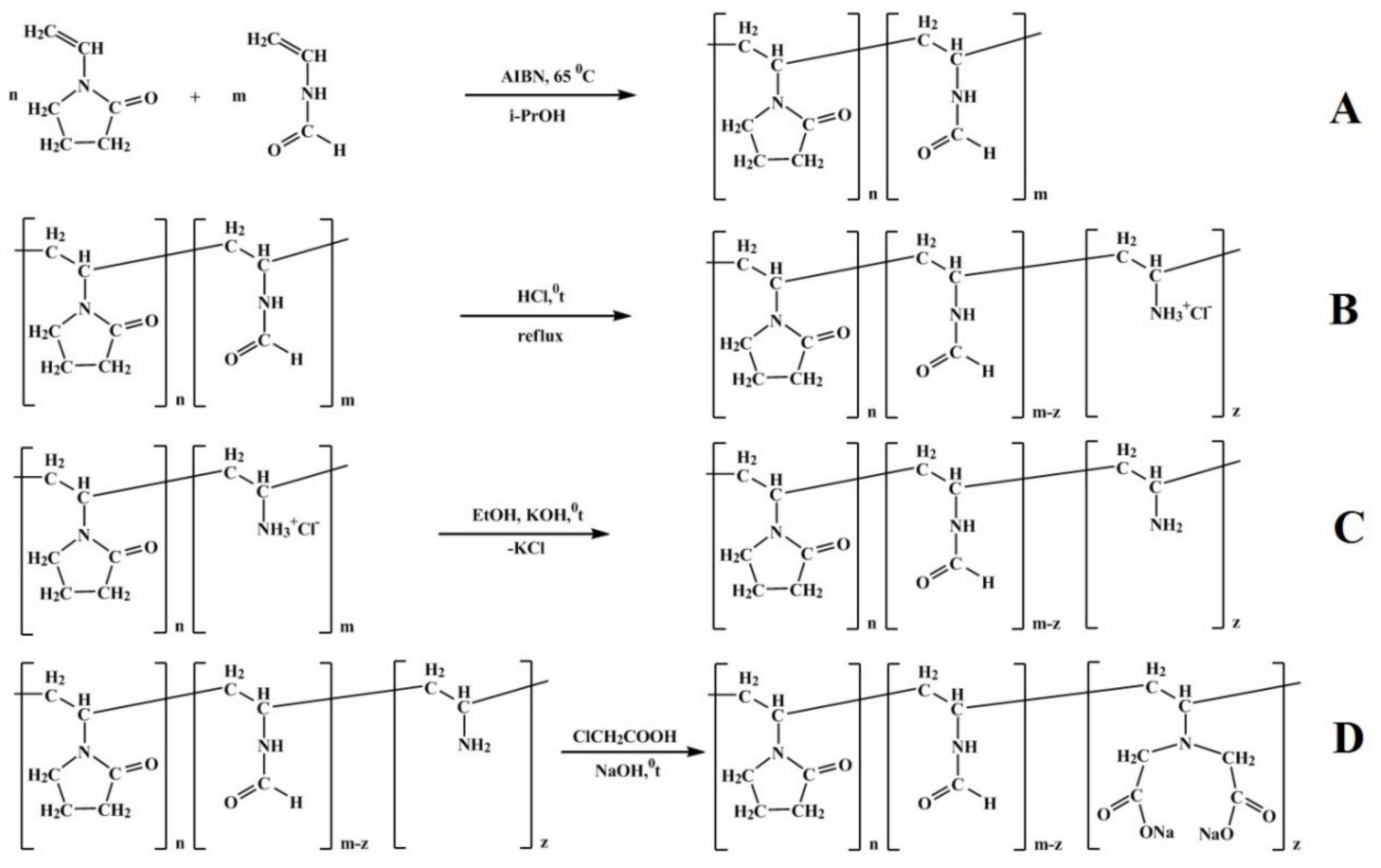
Synthesis of ternary copolymer N-vinylpyrrolidone/N-vinylformamide copolymers containing N-vinyliminodiacetic acid (VP-VFA-VIDA).

Polymerization was carried out in closed vials in argon atmosphere according to the procedure described in [28] (Scheme A, D). Azobisisobutyronitrile (AIBN, 1–2 wt.% with respect to amounts ofloaded monomers) was used as an initiator; the reaction proceeded in ethanol or isopropanol at 60 °С in inert atmosphere for 24 hours. The resulting copolymers were precipitated with diethyl ether. The precipitate was dried at room temperature under vacuum until constant weight was reached. The VP-VFA copolymers were hydrolyzed by 1 M HCl at 90 °С for 7 h; the resulting copolymer (VP-VFA-VA·НCl) was purified by dialysis against distilled water. Degree of hydrolysis was determined argentometrically and from 1H NMR spectra (relative content of signals assigned to VFA fragments). The yield was equal to 90%. Reactions are presented in Scheme 1A,B.

^1^H NMR (500 MHz, D_2_O): δ, ppm,39–1.83 (CH_2_CH(C_4_H_6_NO), br, 2H), 1.84–2.07 (NCH_2_CH_2_CH_2_CO, br, 2H), 2.08–2.50 (NCH_2_CH_2_CH_2_CO, br, 2H), 2.93–3.38 (NCH_2_CH_2_CH_2_CO, br, 2H), 3.41–3.86 (CH_2_CH(C_4_H_6_NO), br 1H) (NHCO, m) 8.0, 7.9.

^13^C NMR (500 MHz, D_2_O): δ, ppm, 17 (NCH_2_CH_2_CH_2_CO), 31(NCH_2_CH_2_CH_2_CO), 35 (CH_2_CH(C_4_H_6_NO), 44 (NCH_2_CH_2_CH_2_CO), 52 (CH_2_CH(C_4_H_6_NO), 163(-NH–CHO), 177 (C=O).

IR (KBr pellets) Lactam C=O 1642 cm^−1^.

M_n_ SEC (0.2 M NaCl) = 42 200 Da, *M*_w_/*M*_n_ = 1.32.

Elementary analysis.

Found: C 18.58%, H 1.21%, N 4.60%.

Calculated: C 18.56%, H 1.25%, N 4.33%

### 2.4. Alkylation of VP-VA Copolymers with ClCH_2_COOH

A calculated amount of 0.5 M NaOH was added to aqueous solution of VP-VA-НCl copolymer (molar ratio 90:10) under stirring; the solution was heated up to 80–90 °C, and then a threefold excess of monochloroacetic acid neutralized with 0.5 M NaOH was added. Then, 5 M NaOH was added so that the pH value reached 10–11. Next, the resulting solution was heated for 6 h, and then allowed to stand overnight at room temperature. Solutions of copolymers were dialyzed against distilled water and freeze-dried. Reactions are presented in Scheme 1C,D.

The copolymer yield was equal to 95%; the composition of the target copolymer (VP: VFA: VIDA) was 90:2:8 (molar ratio); it was estimated from the data of potentiometric titration and ^1^H NMR.

^1^H NMR (D_2_O), δ, ppm,: 3.87, (2, 2′,2″H) (ss), 3.26 (3H), (s), 3.31, 3.22 (5H) (s),1.88, 1.54 (s,s) (1,1′H),1. 53 (1″H).

IR.(KBr pellets), cm^−1^: 1680 υ_as_(COO^−^), 1511, 1459,1445, 1422 υ_s_(COO^−^).

Elementary analysis.

Found. С:55.47%,H: 7,15 %,N: 11.39%

Calculated. С: 55.38%, H: 7.02 %, N:11.47%

Size exclusion chromatography (0.2 M NaCl): *M*_p_ = 42,800 Da, *M*_w_/*M*_n_ = 2.5

### 2.5. Synthesis of Model Complex[Ga(IDA)_2_]Na

A solution of NaIDA (21 mg in 0.5 mL of H_2_O) was added to aqueous solution of GaCl_3_ (10 mg/mL) acidified with HCl (pH = 1). The reaction mixture was stirred for several hours (1–3) at room temperature. Then the solvent was removed under reduced pressure, and the resulting oily product was triturated with diethyl ether. The resulting complex was recrystallized from methanol; the yield was 25 mg (74%). 

^1^H NMR (D_2_O), δ, ppm,dd 3.908, 3.862, 3.834; 3.325, 3.281, 3.231, 3.186. 

IR (KBr pellets), 1672 ʋ_as_(COO), 1500, 1410,1430, 1380 ʋ_s_(COO), 

Elementary Analysis: 

Calculated for [Ga(IDA)_2_]Na_*_10H_2_O: C, 16.55%; H, 3.45%; N, 4.83; 

Found: C, 16.54%; H, 3.48%; N, 4.81.

### 2.6. Histidine Challenge Reaction(HCR)

The HCR was studied as follows. A weighed amount of the polymer (2–3.6 mg) was added to anequimolar ratio of Ga^3+^ in 0.2 М HCl (2 mL), to solution of ^68^Ga (0.5 MBq) in acetate buffer (pH 4.3). In the case or weight amounts of galliumthesolution was neutralized with 1 M NaOH solution until pH = 4. The reaction mixture was kept at room temperature for 1 h, and histidine (tenfold excess) was added.Ga(his)_2_^+^standard complex was prepared by mixing of 0.1 M Ga^3+^ solution in 0.2 М HCl with twofold excess of histidine. Reaction mixture was neutralized up to 6 by adding of 1 M NaOH.

SEC HPLC measurements showed clearly separated peaks of Ga MPC and Ga(his)_2_^+^ with R_t_10.1 and 14.5 min for MPC and standard complex respectively.

### 2.7. Biodistribution Studies

Biodistribution of the MPC in organisms of linear intact laboratory Wistar rats (body weight about 200 g) was studied according to the following protocol. A^68^Ga-VP-VFA-VIDA MPC(43 kDa) (2–3 MBq, 0.25 mL) was injected into the caudal vein. After injection, the animals were sacrificed by decapitation. The target organs, blood (0.5 g), and tissues samples (0.1–0.6 g) and blood samples were placed in tubes with the same geometry. The radioactivity measurements of the ^68^Ga wereperformed using a well-type gamma meter by direct radiometry. The accumulation of the MPC in organs and tissues was calculated as percentage of the total introduced activity per 1 g of an organ/tissue. Since the ^68^Ga radioisotope is short-lived (the half-life is 67.7 min, which is comparable with the time of experiment), this factor was taken into account when recalculating the accumulation in organs and tissues. Prior to measuring the radioactivity of a series of organs and tissues, the radioactivity of the reference sample of the same MPC was measured in the tube of the same geometry. Measurement time of standard sample and selected organs and issue was equal. The measured radioactivity was accepted as the total activity for a specific series taking into account measurement time.

## 3. Results and Discussion

Introducing metal ions into flexible chain polymer carriers is a rather complex task, since coordination properties of polymers depend on (a) molecular mass, polydispersity and structural parameters of polymers; (b) polyelectrolyte effects that exert an influence of conformational behavior of polymers in solutions; and (c) steric availability of coordination groups of BFC [20]. There are also certain limitations that result from peculiarities of hydrolytic behavior of gallium ions in aqueous solutions.

In our previous work [29] it has been demonstrated that introducing macrocyclicpolyligand (1,4,7,10-tetraazacyclododecane-1,4,7,10-tetraacetate (DOTA)) into N-vinylpyrrolidone/N-allylamine copolymer resulted in relatively low radiochemical yields (30–68%) in the reaction between the copolymer and gallium-68.

Iminodiacetic acid (IDA) is a more compact ligand in comparison with DOTA; it forms strong complexes with a majority of metal ions, including gallium (lgК_st_ = 12.76) [30]. In acidic aqueous solutions, coordination number of gallium ion is equal to 6; three coordination sites become filled with donor atoms of BFC, and (IDA)GaX_3_ fragment is formed. The remaining three coordination sites are occupied by labile ligands (X–water, chloride ion), which can be substituted for donor fragments of blood proteins in biologically active media. This substitution may lead to failure in target transport performed by macromolecule. In this work, we assumed that flexible structure of the VP-VA-VIDA copolymer would allow for formation of stable Ga(IDA)_2_ fragments due to intramolecular contacts. In these fragments, all coordination sites of metal ions would be occupied, and the ion would serve as an anchor group between chain fragments. The value of K_st._ should exceed the known values for the most significant BFC forming complexes with gallium ion (i.e., lgК_st. DOTA_ = 21.33 [22]).

In our previous works [31], we have obtained metal-polymer complexes containing indium or radioactive indium-113m and ternary copolymer VP-VFA-VIDA; hydrodynamic characteristics of the complex were virtually similar to those of the initial copolymer carrier. However, the complexes turned out to be unstable in the reaction of interligand exchange with histidine. Ionic radius of gallium is lower than that of indium [32] (0.62 and 0.80 Å, respectively). Possibly, the bite (distance between donor atoms) of VIDA allows for formation of sterically strained fragment In(IDA)_2_, and this fact leads to changes in complex structure during competitive complexation with stronger chelating agents.

Synthesis of copolymers containing N-vinyliminodiacetic acid units was carried out according to Scheme 1. First, radical copolymerization of N-vinylformamide with N-vinylpyrrolidone was carried out according to the procedure described in [28]; then the product was hydrolyzed, and partial alkylation of amino groups with sodium monochloroacetate was performed.

For further studies, we selected the copolymer with a molecular mass equal to 42.8 kDa (calculated from the data of exclusion chromatography, molecular dynamics, and optical studies), which contained 2 mol.% of NH_2_ groups and 8 mol.% of VIDA (according to potentiometric titration). This choice was due to the fact that the copolymer, on the one hand, contains a relatively low amount of BFC, and coordination of gallium ion (followed by formation of intramolecular bonds) should not lead to significant changes in hydrodynamic properties of the macromolecule. On the other hand, its molecular mass facilitates realization of EPR effect (penetration of MPC into cells and retention of the macromolecules inside them). The structure of these copolymers restricts interchain contacts and prevents formation of branched MPC, and amino groups provide conjugation with negatively charged membranes of tumor cells, since the copolymers are polycations.

**Figure 1:**
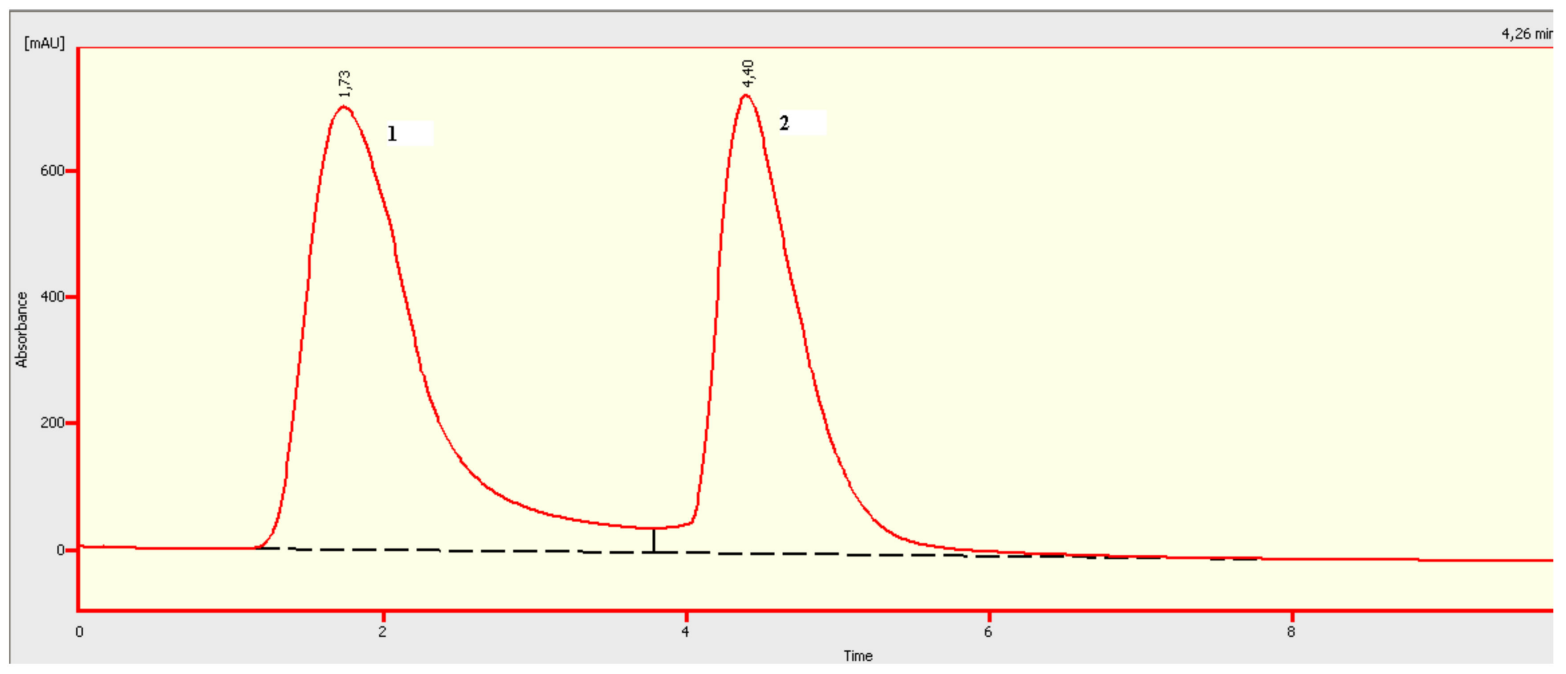
Chromatographic profile of separation of VP-VFA-VIDA (peak 1) and aquatedGa^3+^ (peak 2); reaction time ≈ 2 min; detector wavelength: 220 nm.

Synthesis of gallium MPC was carried out at pH = 4.5 or 5, at ambient temperature, for 20–40 min. In these conditions, gallium ion exists in monohydroxy form (Ga(OH)Сl_2_) and does not form insoluble Ga(OH)_3_. The reaction was monitored by HPLC using ultrashort monolithic columns CIM (convective interaction media) and strong cation exchanger QA (quarterly ammonium). Gradient elution was performed with the use of a system with pH changing from 5.5 (water) to weakly acidic pH = 2 (0.01 N HCl). The use of monolithic sorbents provides for fast (5–10 min) efficient separation of MPC/metal ion mixture (Figure 1). Unlike the sorbents used in traditional exclusion chromatography, the structure of monolithic sorbent modified with cation exchangers excludes sorption of macromolecules and metal ions on its surface, while metal hydroxides of various compositions become irreversibly adsorbed on column filler. It should be noted that the used chromatographic system is convenient under conditions of radiochemical synthesis due to its express operation and possibility of repeated use of the column without the need for washing out adsorbed ^68^Ga.

The selected system makes it possible to separate the initial water-soluble polymers, MPC, and non-reacted aquated metal ion. Macromolecular components are eluted with virtually zero volume, and non-reacted Ga(H_2_O)_6_^3+^ion has a retention time (Т_r_) of 4.7 min.

Molecular masses and related parameters were determined by size exclusion liquid chromatography (SEC) in 0.2 M solution of NaCl. The results are given in Table 1. In calculations of weight-average (*M*_w_) and number-average (*M*_n_) molecular masses and polydispersity index *M*_w_/*M*_n_, we used the Mark–Kuhn–Houwink (MKH) constants for poly(N-vinylpyrrolidone) and poly(N-vinylformamide) [28].

UV spectrum of the synthesized model complex Ga(IDA)_2_Na has the single characteristic absorption maximum in the area of 210–230 nm that is attributed to charge transfer from metal to ligand. Therefore, this wavelength region was used in all HPLC measurements.

It was established that at the Ga^3+^: [VP-VFA-VIDA] ratio equal to 1:1, metal ion was completely bound to the copolymer within 20–40 min at room temperature.

In order to confirm coordination between metal ion and chelating site, metal-polymer complex VP-VFA-VIDA-Ga^3+^ was studied by IR and ^1^Н NMR spectroscopy.

**Figure 2:**
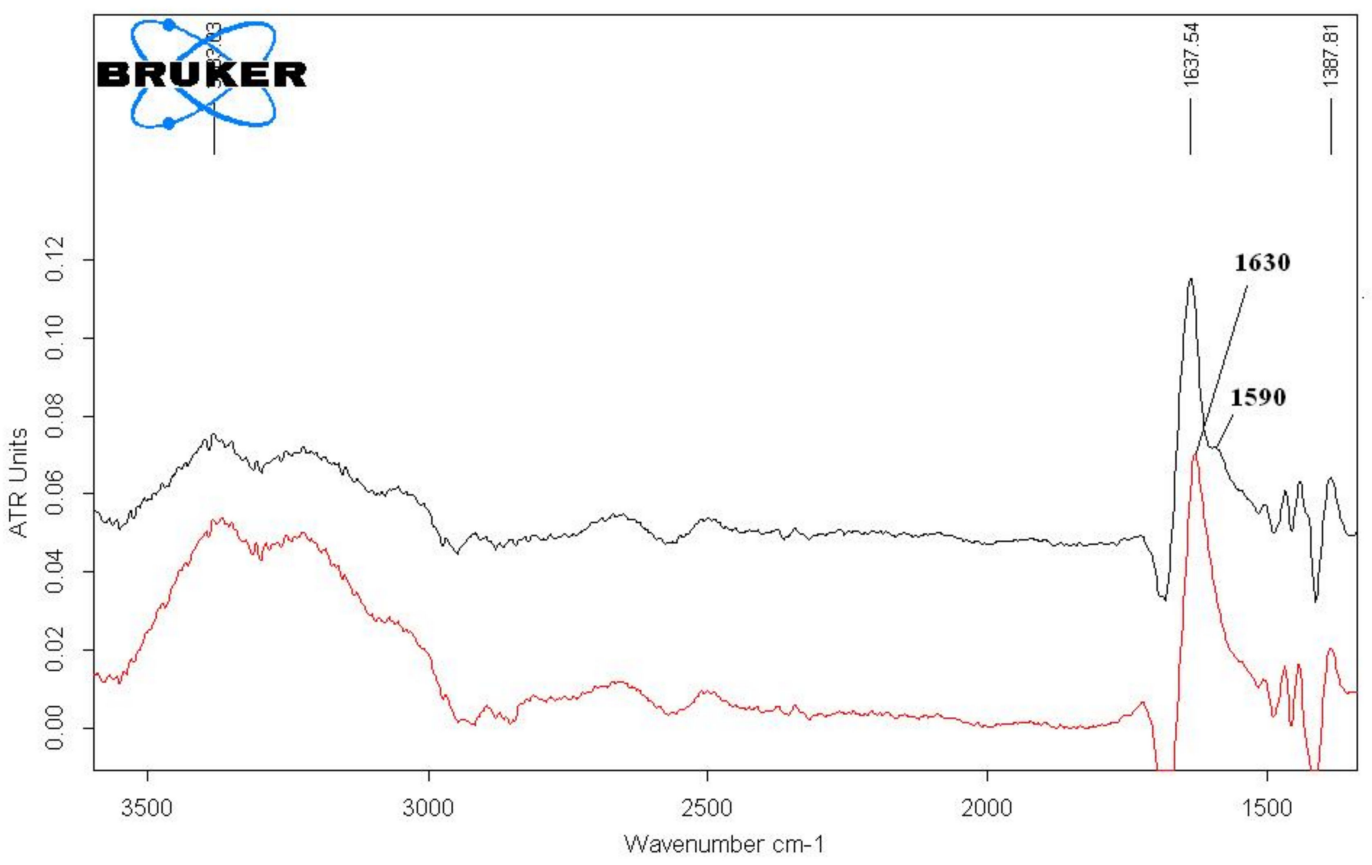
IR spectra of VP-VFA-VIDA (lower spectrum) and VP-VFA-VIDA–Ga metal-polymer complexes ((MPC (upper spectrum)) after subtraction of the initial copolymer spectrum.

The most intensive band in IR spectra of MPC is the one assigned to vibrations of carbonyl groups (υ_as(С=О)_) about 1650 cm^−1^; it is overlapped with the peak attributed to vibrations of C=O fragments in lactam rings of N-VP units. After subtraction of the spectrum of the initial copolymer from the spectrum of MPC, we were able to reveal the characteristic band of υ_as(СOO_^-^ vibration (1590 cm^−1^), which was shifted toward lower frequencies; this fact indicates formation of a metal-ligand bond (Figure 2).

**Figure 3:**
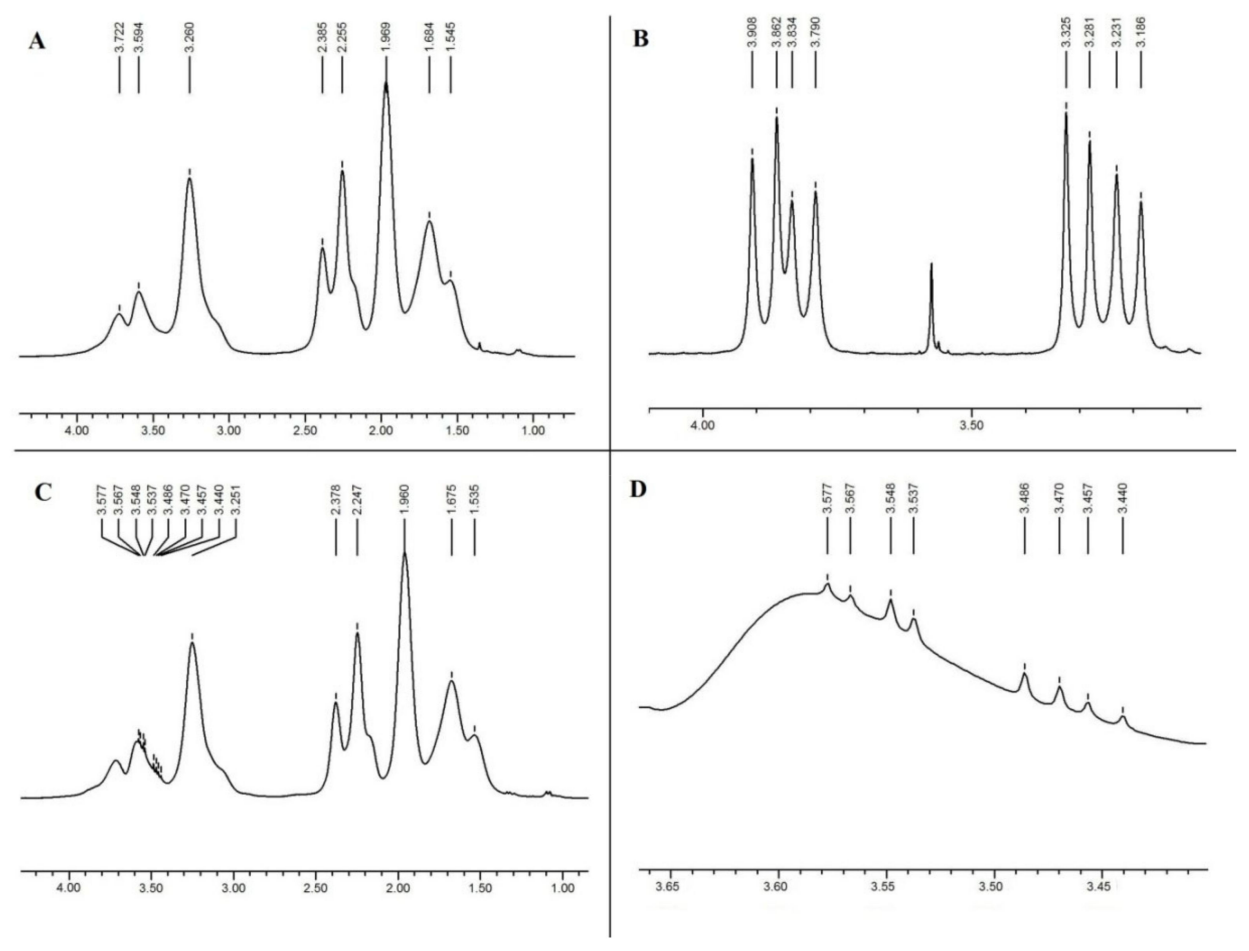
^1^Н NMR spectra (D_2_O): (а) the initial copolymer VP-VFA-VIDA; (**A**) VP-VFA-VIDA-Ga^3+^; (**B**) spectrum of the model complex Na[Ga(IDA)_2_]; (**C**,**D**) signals of 2, 2″6, 6′ 6, 6″, 6 protons.

It is seen in the ^1^Н spectrum of VP-VFA-VIDA copolymer (Figure 3A) that the singlet peak produced by СН_2_ protons of IDA ligand is overlapped with more intensive signals assigned to 2, 2′, 2″ protons of polymer backbone. After coordination between metal ion and IDA fragment of the copolymer, these protons become non-equivalent, and manifest themselves in the spectrum as a group of doublet signals in the area around 3.8 and 3.2 ppm (Figure 3C,D). This effect is clearly seen in the spectrum of the model complex Ga(IDA)_2_Na (Figure 3B).

With the purpose of accurate determination of molecular masses, molecular mass distributions and hydrodynamic parameters of the initial copolymer (VP-VFA-VIDA) and its MPC, these objects were studied by hydrodynamic and optical methods.

Table 2 gives the values of the following parameters obtained for the initial copolymers and MPC: intrinsic viscosity [*η*], translational diffusion coefficient *D* and sedimentation coefficient *s* in 0.2 М NaCl (24 °С), molecular masses *M*_sD_, hydrodynamic radii *R*_h_ (determined from the DLS data), refractive index increments (*dn*/*dc*), and the values of the Tsvetkov–Klenin hydrodynamic constant calculated according to the formula [33] *А*_0_ = (*η*_0_*D*/*T*) × ([*η*]·*M*_sD_)^1/3^.

It is seen that molecular masses of the initial copolymers and metal-polymer complexes are similar. Besides, the values of [*η*] for the initial copolymers obtained in water solution, 0.2 M NaCl, and benzyl alcohol also coincide within the measurement accuracy. In this case, the Flory relationship [*η*] = *Ф*$\bar{\left(h^{2}\right)}$^3/2^/*M* [34] gives us reason to believe that root-mean-square sizes of macromolecular coils $\bar{\left(h^{2}\right)}$ of the copolymer and its complexes with gallium are similar in various solvents. In other words, the equilibrium rigidity parameter (the Kuhn segment *А* = $\;\bar{\left(h^{2}\right)}$/*L*, where *L* is the polymer length) for these systems also remained unchanged.

Molecular parameters of MPC after storage in dry state for a prolonged period of time (from 1(2) to 24 months) were studied again in 0.2 M NaCl. It is seen from Table 2 that molecular masses remained unchanged, although sizes of molecules ([*η*] and *R*_h_) decreased insignificantly (~5%). This fact indicated high thermodynamic stability of the formed MPC.

The data concerning hydrodynamic radii *R*_h_ (Table 2) also do not reveal any noticeable differences between the values for the initial copolymer (*R*_h_ = 8.9 nm) and MPC (*R*_h_ = 7.5 nm for its complex with gallium). Taking into account measurement inaccuracy (~5%), we can conclude that MPC size decreases only slightly (~15%) as compared to that of the pure copolymer. This difference can be attributed to possible compactization of MPC due to formation of intramolecular crosslinks involving Ga(IDA)_2_ fragments.

**Scheme 2:**
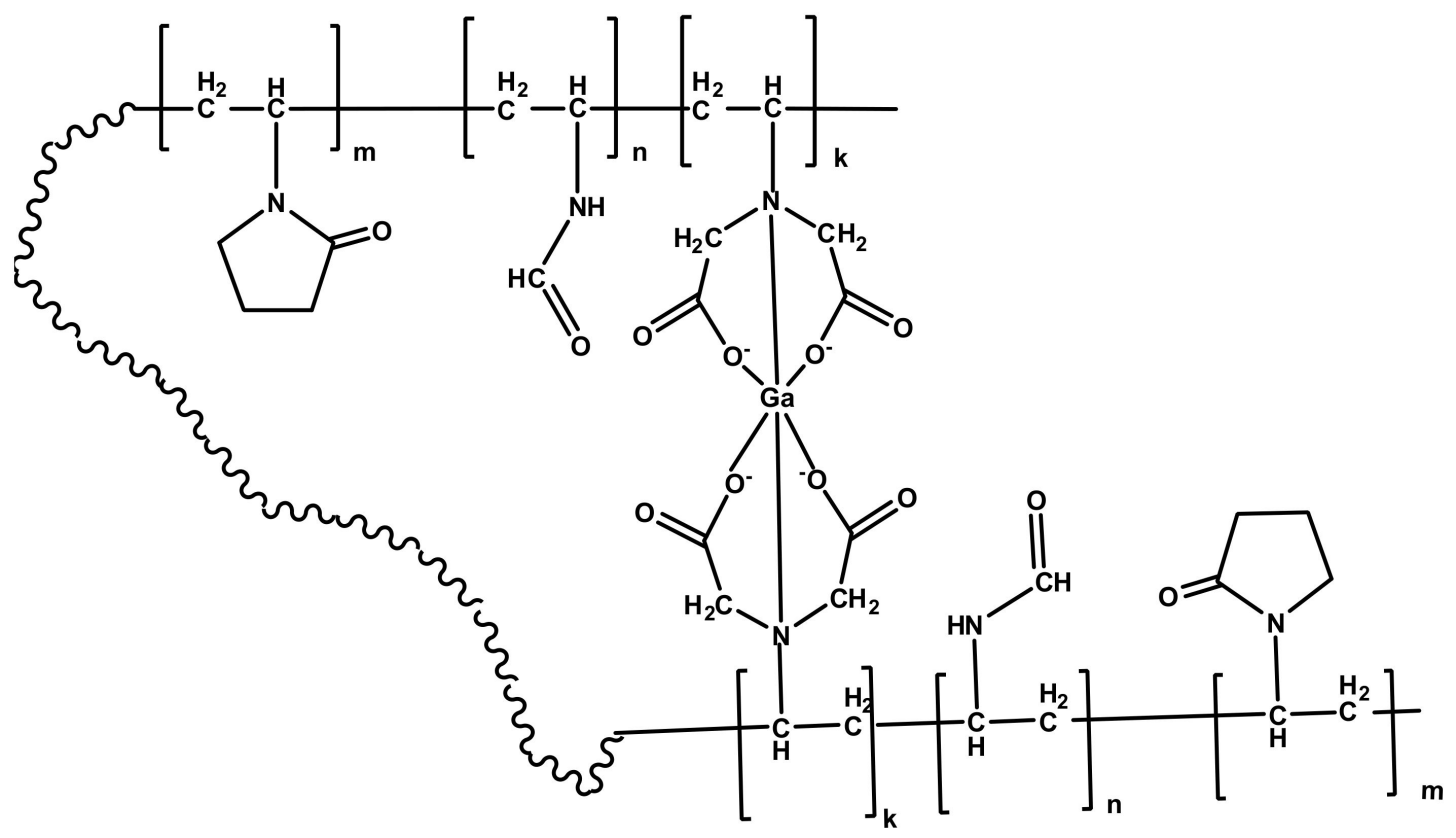
Proposed structure of MPC Ga-VP-VFA-VIDA.

Thus, a proposed structure of MPC Ga-VP-VFA-VIDA could be presented as follows (Scheme 2).

The results of hydrodynamic and optical studies indicated that addition of low amounts of Ga(H_2_O)_6_^3+^ ion as a complexating agent did not result in any conformational or structural changes in going from the copolymer to its complexes; formation of interchain complexes did not occur.

We should also take into account the fact that only 8% of monomer units of the macromolecule take part in complex formation. If any conformational or structural transformations possibly take place in going from the copolymer to its complexes, they are very insignificant, and changes in optical properties of the objects remain within measurement error.

**Figure 4:**
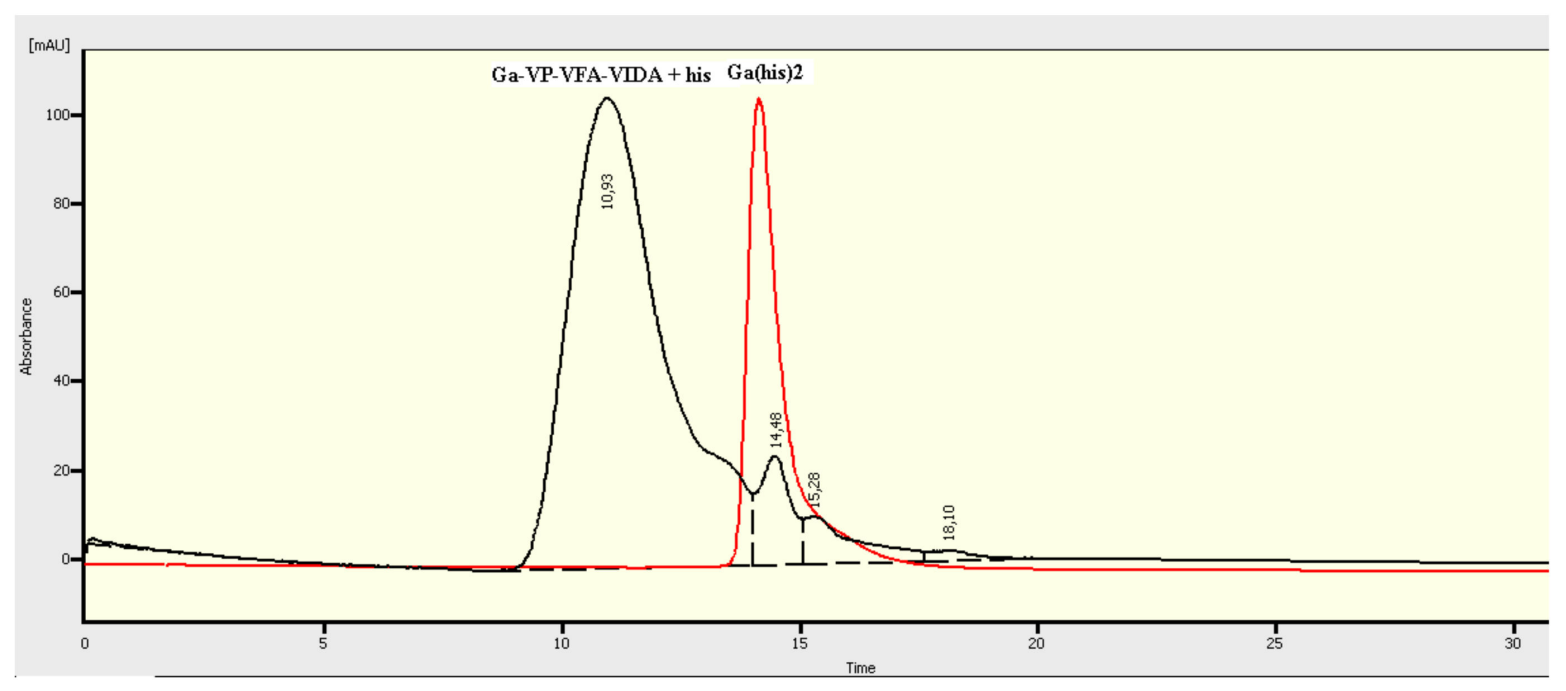
SEC profiles of conjugate between VP-VFА-VIDA-Ga and histidine, and the model complex Ga(His)_2_^+^. Column: ultrahydrogellinear 0.78×30 cm, 0.6×4 cm, eluent: 0.2 М NaCl; elution rate: 0.8 mL/min.

Stability of VP-VFA-VIDA-Ga metal-polymer complex was estimated by size exclusion chromatography at various pH values (1–8); its high stability against hydrolysis was demonstrated. Besides, we studied stability of VP-VFA-VIDA-Ga in the presence of histidine (the so-called histidine challenge reaction (HCR)), which is one of the most important amino acids and a strong chelating agent for a majority of metal ions. MPC did not undergo noticeable changes; at the same time, a small amount of bis-chelate complex Ga(His)_2_^+^ (3%) appeared. The VP-VFA-VIDA-Ga complex was stable in the presence of histidine at pH=7 for 3–4 h, which is quite acceptable for biological tests (Figure 4).

### 3.1. Radiochemical Synthesis of Metal-Polymer Conjugates VP-VFA-VIDA-^68^Ga

Radiochemical synthesis of MPC was carried out in aqueous solutions at рН = 5.5. The course of the reaction was monitored by the HPLC technique described above. Copolymer concentrations in the 20–80 µg/mL range were determined; it was demonstrated that the maximum yield of isotope-containing complex (85–90%) was observed at a polymer concentration of 80 µg/mL.

Non-reacted ^68^Ga^3+^ was removed using a semi-automatic purification system equipped with the cartridge containing Sephadex-25 Superfine sorbent.

**Figure 5:**
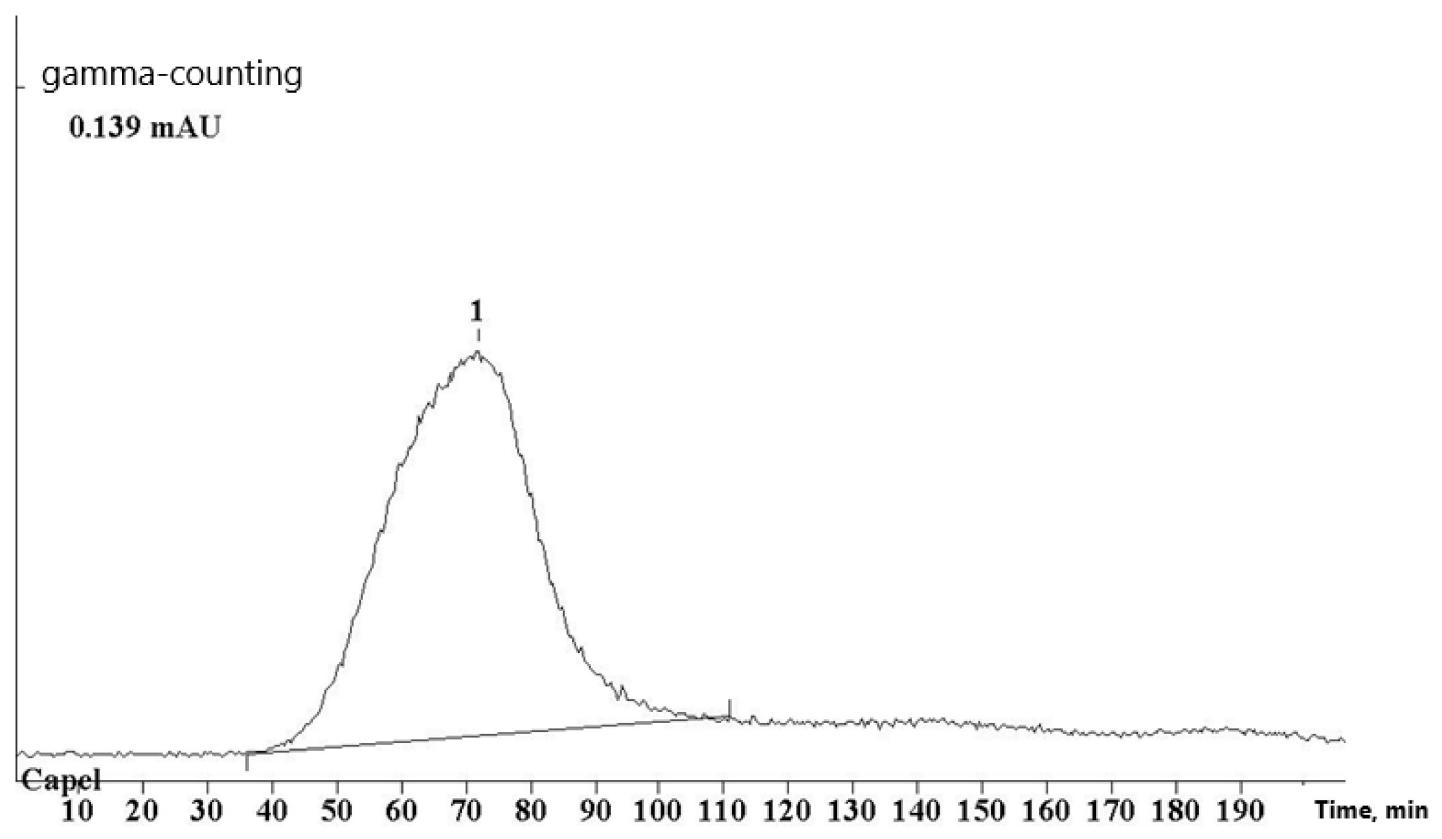
Isolation of VP-VFA-VIDA-^68^Ga with Sephadex G-25 superfine sorbent by gel filtration. Sample volume: 0.5 mL; eluent: 0.9% NaCl; time, min. Radiochemical yield of the target product: 80% (calculated using Equation (1)). Peak 1: VP-VFA-VIDA-^68^Ga.

A representative chromatographic profile obtained in the course of isolation of the MPC [VP-VFA-VIDA-^68^Ga] (radioactivity detection) is given in Figure 5. It is seen that MPC of gallium-68 is eluted with an approximate volume of V_о_, while gallium ions are virtually completely adsorbed on the cartridge filler (according to the measurements of cartridge residual activity). The time of isolation of the target product was 5–7 min, which entirely complies with conditions of radiochemical synthesis and meets the requirements for work with short-lived isotope gallium-68.

High yield of MPC after purification with the cartridge, and its rather high specific activity (15 mBq/mL) fully complies with conditions of experiments involving laboratory animals and the studies of biodistribution of metal-polymer complex of ^68^Ga.

Radiochemical yield (RCY) of complexation reaction was calculated according to the following Equation:

RCY = 100% × А_о_/(А_о_ + А_е_ + А_col_.)

where *А*_о_is the activity of the *V*_о_ fraction after correction for decay occurring by the time when the sample is introduced into the column. *А*_е_ is the activity of the *V*_e_ fraction after correction for decay occurring by the time when the sample is introduced into the column; *А*_col._ is the activity of the column after correction for decay occurring by the time when the sample is introduced into the column.

Radiochemical purity of the complex was additionally examined by size exclusion chromatography and found to be 98% (ultrahydrogellinear 0.78 × 30 cm, 0.6 × 4 cm; eluent: 0.2 М NaCl; elution rate: 0.8 mL/min).

Since molecular mass of VP-VFA-VIDA-^68^Ga is almost similar to that of albumin (the main protein component of blood plasma), it turned out to be impossible to study stability of the metal-polymer complex in vitro. In order to investigate its stability in biologically active media, we studied interaction between the complex and histidine. Similarly to the case of non-radioactive analog, these experiments showed high stability of MPC containing ^68^Ga.

### 3.2. Biodistribution Studies

**Figure 6:**
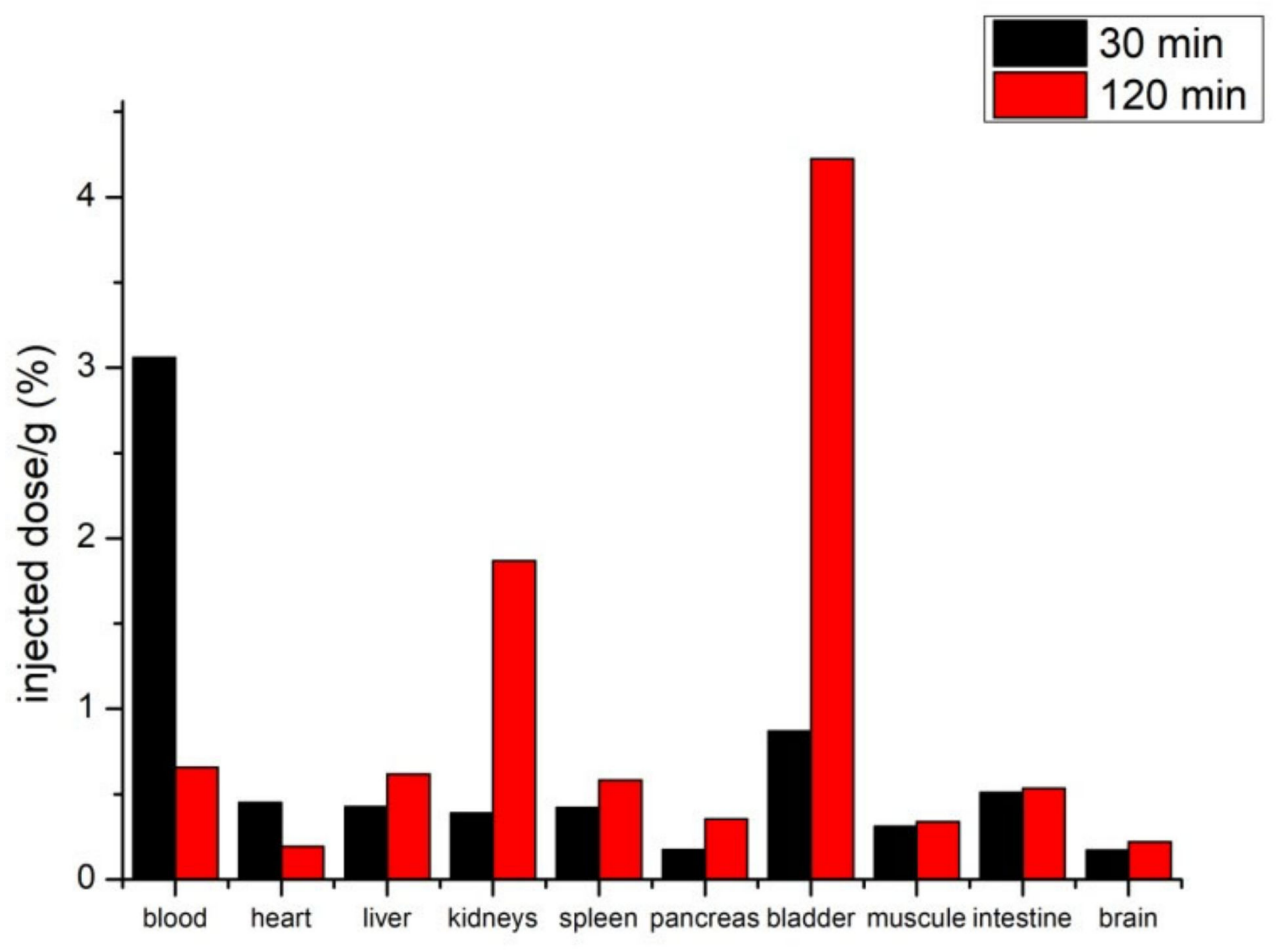
Biodistribution of the VP-VFA-VIDA-^68^Ga complex in intact Wistar rats (200 g), injected with 2–3 MBq and sacrificed after 30 and 120 min.

Biodistribution of MPC VP-VFA-VIDA-^68^Ga (MM = 43 kDa) in intact laboratory animals (Wistar rats) was investigated (Figure 6). It is seen from the presented data that in the case of intact animals, the active substance is primarily accumulated in bloodstream (3.05% of the injected dose per 1 g of a tissue (ID/g (%)) and in urinary tract (kidney (1.86%) and bladder (4.23%)). It is noteworthy that the active substance is rather rapidly (in 120 min after injection) excreted through the urinary tract (kidney (0.39%) and bladder (0.86%)). Noticeable accumulation of the radioactive substance in liver was also observed ((0.43% (30 min), (0.62% (120 min)).

Due to its structural and molecular characteristics, the synthesized copolymer labeled with gallium-68 can be regarded as a promising basic substance for development of affine VP-based macromolecules decorated with target vectors (for instance, short peptides) for binding with specific tumor sites.

## 4. Conclusions

The results obtained during physico-chemical, chromatographic and spectroscopic studies of the synthesized VP-VFA-VIDA copolymers indicate formation of unimolecular complex between the copolymer chain and Ga^3+^ ion. In this complex, metal ion acts as an anchor group between VIDA units of the same polymer chain. The obtained MPC are thermodynamically stable and also stable in histidine challenge reaction. Hydrodynamic radius of MPC (*R*_h_ = 7–7.5 nm) is quite suitable for realization of EPR effect [8]. Sufficiently high radiochemical yield of the target MPC and its specific activity were revealed. In vivo experiments demonstrated high accumulation of the MPC in blood in 30 min after injection, its relatively rapid clearance via urinary tract, and decrease in the content of radioactive compound in bloodstream in 120 min after injection.

## Figures and Tables

**Table 1:** Molecular masses and polydispersity characteristics of VP-VFA-VIDA copolymer and metal-polymer complex VP-VFA-VIDA-Ga^3+^ determined by size exclusion chromatography.

Sample	Retention Time, min	*М*_р_, Da	*М*_n_, Da	*М*_w_, Da	*M*_w_/*M*_n_ (PDI)
VP-VFA-VIDA	10.27	42,800	24,700	62,300	2.5
VP-VFA-VIDA-Ga^3+^	10.43	44,200	25,300	53,300	2.1

*R*_T_ is the retention time; *M*_p_ is the retention time at peak maximum; *M*_n_ is the number-average molecular mass; *M*_W_ is the weight-average molecular mass; *M*_w_/*M*_n_ is the polydispersity index.

**Table 2:** Molecular characteristics of the initial copolymer and MPC in 0.2 М NaCl solution calculated using the value of hydrodynamic invariant *А*_0_.

Sample	[*η*], dL/g	*D*⋅10^7^, cm^2^/c	S, Sv	*M*_sD_	*R*_h_, nm	*dn*/*dc*	*А*_0_⋅10^10^, Erg/К
VP-VFA-VIDA	0.35	4.8	1.9	43,000	8.9	0.14	4.0
VP-VFA-VIDA-Ga	0.35	4.6	-	43,000	7.5	0.14	-
VP-VFA-VIDA-Ga (2 years later)	0.29	5.8	2.2	41,500	7.0	0.15	4.4
